# Optimism and mental health in college students: the mediating role of sleep quality and stress

**DOI:** 10.3389/fpsyg.2024.1403146

**Published:** 2024-07-16

**Authors:** Yun-Ju Lai, En-Yun Tsai, Ploypapus Jarustanaput, Yi-Syuan Wu, Yi-Hau Chen, Samantha E. O’Leary, Sumatchara Manachevakul, Yuan Zhang, Jiabin Shen, Yan Wang

**Affiliations:** ^1^School of Nursing, Zuckerberg College of Health Sciences, University of Massachusetts Lowell, Lowell, MA, United States; ^2^School of Pharmacy, College of Medicine, National Taiwan University, Taipei, Taiwan; ^3^Institute of Biomedical Sciences, Academia Sinica, Taipei, Taiwan; ^4^Institute of Statistical Science, Academia Sinica, Taipei, Taiwan; ^5^Department of Psychology, College of Fine Arts, Humanities & Social Sciences, University of Massachusetts Lowell, Lowell, MA, United States

**Keywords:** optimism, sleep quality, stress, anxiety, depression, mediation analysis

## Abstract

**Objective:**

College students showed a high prevalence of stress, anxiety, and depression, with medical and nursing students experiencing particularly elevated levels of mental health challenges.

Optimism significantly influences overall well-being by promoting a healthy lifestyle and cognitive responses. However, the association of optimism with sleep quality, stress, and mental health in college students remains unexplored. This study aimed to (1) explore the associations of optimism with sleep quality, stress, and mental health and (2) ascertain whether sleep quality and stress mediate the association between optimism and mental health among college students.

**Methods:**

A cross-sectional study was conducted using online surveys with students from health science majors at a public university in the northeast United States from September to December 2022. A total of 222 students participated in the study, providing data on sociodemographics, optimism, sleep quality, stress, anxiety, and depression. Parallel and serial mediation models were utilized to examine the potential mediating roles of sleep quality and stress in the association between optimism and mental health.

**Results:**

The study found that optimism influences anxiety and depression through both direct and indirect pathways. In line with predictions, the parallel mediation analysis revealed that the impact of optimism on anxiety (β_total_ = −0.598, 95% confident interval [CI]: −0.778 to −0.392) and depression (β_total_ = −0.724, 95% CI: −0.919 to −0.519) was mediated by stress and sleep quality. Furthermore, the serial mediation models revealed that stress and sleep quality co-mediated the relationship betweenoptimism and anxiety (indirect effect [IE] = −0.074, 95% CI: −0.135 to −0.029) or depression (IE = −0.084, 95% CI: −0.142 to −0.036) in a sequential manner.

**Conclusion:**

Optimism was negatively correlated with poor sleep quality, stress, anxiety, and depression. Enhanced optimism was linked to high sleep quality and less stress, anxiety, and depression. These insights emphasize the potential for school-based optimism interventions to improve sleep quality, ameliorate stress-related concerns, and alleviate mental health challenges in college students.

## 1 Introduction

Stress, anxiety, and depression are increasingly problematic in our society, with severe consequences for both physical and mental health (Blanco et al., 2021). Beyond personal health implications, mental health problems impose a substantial economic burden on society, projected to reach around 16 trillion US dollars for the global economy by 2030 (Trautmann et al., 2016; Patel et al., 2018). College students are a particularly vulnerable population, exhibiting a higher prevalence of mental health issues compared to the general population (Mofatteh, 2021). According to the National College Health Assessment from the American College Health Association, over 76% of undergraduates reported moderate or severe psychological distress (American College Health Association, 2022). Furthermore, in the National Healthy Minds Study, 41% of college students disclosed their experience of major and moderate depression and 36% of them reported moderate or severe anxiety (Healthy Minds Network, 2023). Poor mental health in college students may be associated with a range of problems, such as impaired academic performance, heavy alcohol consumption, substance abuse, low self-esteem, and suicide attempts (Hysenbegasi et al., 2005; Skidmore et al., 2016; Hiçdurmaz et al., 2017; Liu et al., 2019; Sæther et al., 2019). Given these costly outcomes for students, universities, and society, the mental health of undergraduate students is not only a crucial public health concern but also a pressing research priority.

College students’ poor mental health may stem from various challenges, including concerns regarding academic performance, pressure to succeed, and post-graduation plans (Beiter et al., 2015). Particularly, students in health science fields such as medicine and nursing often grapple with heightened levels of anxiety and depression than other non-medical peers due to their heavy workload, including theoretical responsibilities and hands-on patient care (Mofatteh, 2021). In addition, the quality of sleep has been closely linked to mental health issues, with poor sleep quality exacerbating the susceptibility of college nursing students to mental illnesses, including anxiety and depression (Zhang et al., 2018). Students suffering from poor sleep quality often confronted high levels of perceived stress, which in turn precipitated anxiety or depression symptoms (Doane et al., 2015; Zhang et al., 2018), impaired psychosocial functioning (Tavernier and Willoughby, 2014), and negatively impacted academic performance (Okano et al., 2019). Furthermore, individuals with heightened stress were prone to develop concurrent anxiety (Ghorbani et al., 2008), exhibit compromised sleep quality (Liu et al., 2017), employ less healthy coping strategies (Evans et al., 2015), and thus manifest depressive symptoms.

Optimism, defined as harboring positive expectations for the future (Scheier and Carver, 1985), is a positive personality trait contributing to positive psychology (Seligman and Csikszentmihalyi, 2014). Optimism is particularly vital during periods of uncertainty (Carver et al., 1989), as demonstrated by its role as a protective factor against fear, stress, anxiety, and depression during the COVID-19 pandemic (Vos et al., 2021). Heightened levels of optimism are associated with lower levels of anxiety and improved academic achievement among college students (Singh and Jha, 2013), as well as better coping skills in response to stress (Solberg Nes et al., 2009). Recent studies have demonstrated that optimism can promote positive emotions and higher life satisfaction, particularly during the COVID-19 pandemic (Martinez et al., 2022). On the contrary, some studies suggested that lower levels of optimism and hope are aligned with decreased subjective well-being among young adults facing high levels of stress due to the pandemic (Genç and Arslan, 2021).

In the present, with the majority of studies focusing on the psychological health problems in college students, the mechanisms implicated in the links among optimism, sleep quality, stress, and mental health remain unclear. Therefore, this study aimed to Blanco et al. (2021) explore the associations of optimism with sleep quality, stress, and mental health; and Trautmann et al. (2016) ascertain whether sleep and stress mediate the connection between optimism and mental health, providing valuable insights into a promising intervention strategy regarding elevating optimism levels, thereby bolstering students’ mental well-being as they confront adversity.

## 2 Materials and methods

### 2.1 Study design and setting

This study was a quantitative cross-sectional study conducted among 222 undergraduate students in health science majors at a public university in the northeast United States in the fall of 2022. We employed a non-probability purposive sampling method to administer online surveys to all freshmen, sophomore, junior, and senior health science students.

### 2.2 Inclusion and exclusion criteria

We included undergraduate students who were: Blanco et al. (2021) aged 18 years or older; Trautmann et al. (2016) currently enrolled full-time; and Patel et al. (2018) having access to the internet and capacity in computer typing. Students who were unable to provide informed consent were excluded.

### 2.3 Sample size

A minimum correlation coefficient of 0.3 was assumed for variables including optimism, sleep quality, stress, and mental health, as supported by previous studies (Cohen, 2013; Zhang et al., 2018). With an aim to achieve 80% statistical power and a type I error rate of 0.05, a power analysis suggested a sample size of at least 67 participants for this study.

### 2.4 Measurements

#### 2.4.1 Revised life orientation test

The LOT-R was employed to measure the level of optimism (Scheier et al., 1994). Consisting of 10 items, the LOT-R presents with 3 items, respectively, oriented in positive and negative directions, along with 4 filler items. Respondents are requested to indicate the extent to which they agree with each item on a 5-point Likert scale that ranges from strongly disagree to strongly agree. The total score is from 0 to 24, and the higher the LOT-R score, the higher the levels of optimism. The acceptable internal consistency of the LOT-R was reported as Cronbach’s α of 0.82 (Shifren and Anzaldi, 2018) in the stroke population, and stability (test–retest reliability) over a 4-month period in college students was reported as *r* = 0.79 (Scheier et al., 1994). A scoring range of 0 to 13 points indicates a low level of optimism, whereas a range of 14 to 18 points suggests moderate optimism, and 19 to 24 points signifies a high level of optimism (Przybyszowski et al., 2022). In this study, the scale demonstrated good reliability with a Cronbach’s alpha of 0.79.

#### 2.4.2 Pittsburgh sleep quality index

The PSQI was used to assess the sleep quality in the previous month (Buysse et al., 1989). Comprising 19 questions concerning sleep habits, the PSQI involves 7 components: sleep duration, sleep latency, sleep disturbance, daytime dysfunction, use of sleeping medications, habitual sleep efficiency, and overall sleep quality. This instrument has been previously validated for college students (Lemma et al., 2014). Each component is scored on a scale of 0 to 3, yielding a global sleep quality score ranging from 0 to 21. Participants with PSQI scores exceeding 5 were identified as experiencing poor sleep quality (Buysse et al., 1989). The PSQI in this study demonstrated good reliability with a Cronbach’s alpha coefficient of 0.63.

#### 2.4.3 Perceived stress scale

The PSS is a 10-item questionnaire for evaluating the perception of stress during the past month (Cohen et al., 1983). Participants were asked to rate the frequency of experiencing certain feelings and thoughts using a 5-point Likert scale. With 4 positively stated items reverse scored (e.g., 0 = 4, 1 = 3, 2 = 2, 3 = 1, and 4 = 0), the total PSS scores range from 0 to 40 and the higher PSS scores indicate the higher level of stress. The scale demonstrated good internal consistency (Cronbach’s alpha) previously in undergraduate students (Lin et al., 2020). The Cronbach’s alpha in this study was 0.81.

#### 2.4.4 General anxiety disorder-7

The GAD-7 is a self-administered 7-item questionnaire, designed to assess the symptom severity of anxiety (Spitzer et al., 2006). The GAD-7 is an acceptable questionnaire, with Cronbach’s alpha of 0.89 in general populations (Lowe et al., 2008). Each item is scored on a 4-point Likert-type scale, ranging from 0 to 3, and summed up with a final score ranging from 0 to 21. The scores of 5, 10, and 15 are cutoff points indicating mild, moderate, and severe levels of anxiety (Spitzer et al., 2006). The GAD-7 scale demonstrated excellent reliability with a Cronbach’s alpha of 0.92 for this study sample.

#### 2.4.5 Patient health questionnaire 9

The PHQ-9 is a self-administered questionnaire, employed to test the extent of depression (Kroenke et al., 2001). The internal reliability of the PHQ-9 was demonstrated, with Cronbach’s alpha of 0.81 to 0.84 (Kroenke et al., 2016). Composed of 9 items, each rated on a 4-point Likert-type scale ranging from 0 to 3, the total scores on the PHQ-9 range from 0 to 27. Cutoff scores of 5, 10, 15, and 20 represent mild, moderate, moderately severe, and severe depression (Kroenke et al., 2016). In this study, the scale demonstrated excellent reliability with a Cronbach’s alpha of 0.90.

### 2.5 Data collection

Structured questionnaires comprising students’ sociodemographics (e.g., age, biological sex, race/ethnicity, study majors, grade levels, and history of psychotherapy, psychiatric medication, or psychiatric disorder), optimism (LOT-R), sleep quality (PSQI), stress (PSS), anxiety (GAD-7), and depression (PHQ-9), were administered through Qualtrics Online Surveys. Within the online survey, all the subjects were introduced to the study’s purpose and procedures, potential risks and benefits, and assurance of privacy and confidentiality before proceeding to the survey sections. Additionally, embedded consent in the online survey required participants to agree prior to the initiation of the survey.

### 2.6 Data analysis

Following the assessment of statistical normality, descriptive statistics, including the number of participants, age, biological sex, race/ethnicity, study majors, and grade levels, were reported as mean ± SD or frequency (%). The Pearson correlation was applied to the relationships between sleep quality, stress, and mental health among college students. All the analyses were performed using SPSS 28.0 for Windows (SPSS Inc., Chicago, IL). Values of *p* < 0.05 were considered statistically significant. Mediation analysis, for both parallel and serial mediating effects of stress and sleep quality on the relationship between optimism and mental health, was performed using the package lavaan (Rosseel, 2012), version 0.6.16, implemented in the R system for statistical computing (Team RC, 2013). Mediation models were adjusted for the potential covariate, biological sex, to statistically control its effects and more accurately estimate the relationship between the predictors and the outcomes in the models.

## 3 Results

### 3.1 Participant characteristics

**Figure 1:**
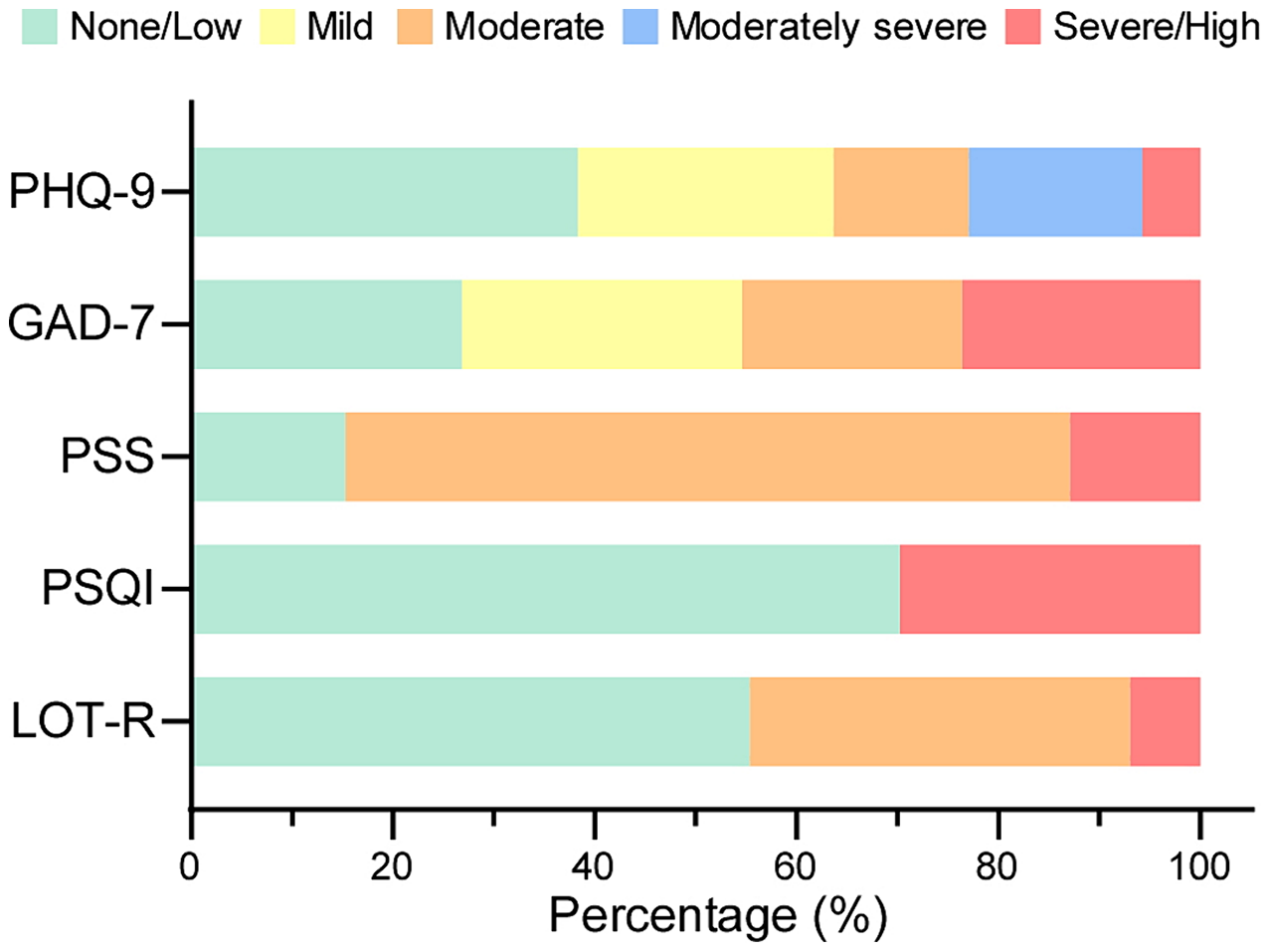
The proportion of students across various severity levels in PHQ-9, GAD-7, PSS, PSQI, and LOT-R. The X-axis indicates the percentage of students, and the Y-axis denotes the variable measurements. LOT-R, revised Life Orientation Test; PSQI, Pittsburg Sleep Quality Index; PSS, Perceived Stress Scale; GAD-7, General Anxiety Disorder-7; PHQ-9, Patient Health Questionnaire-9.

A total of 222 students participated in the study, with a mean age of 20.3 (± 2.44) years. Predominately, respondents were female (81.5%), nursing (40.5%), sophomore (33.8%), and junior (31.1%) students. Almost 20% of the participants had a psychiatric history, with 59.5 and 50.0% of them experiencing anxiety and depression, respectively (Table 1). Figure 1 provides an overview of variable measurements within the respondent population. The LOT-R scores across the study population indicated a range of low optimism with a mean score of 13.12 ± 3.99. It is noteworthy that 55.3% of the students were categorized as having low optimism, while 37.7% fell into the moderate optimism category (Table 2). As outlined in Table 2 and Figure 1, participants reported experiencing various psychological states, including poor sleep quality, moderate stress, mild anxiety, and mild depression.

**Table 1:** Descriptive statistics for respondent demographic characteristics.

Characteristics	Subjects
Age, year, Mean (S.D.)	20.3 (2.44)
Sex, n (%)	
Male	41 (18.5)
Female	181 (81.5)
Race/ethnicity, n (%)	
Non-Hispanic White or European-American	97 (43.7)
African American, Afro-Caribbean	30 (13.5)
Latino or Hispanic American	43 (19.4)
Asian and Asian American	50 (22.5)
Others	2 (0.9)
Study Major, n (%)	
Biomedical Sciences	37 (16.7)
Exercise Science	29 (13.1)
Nursing	90 (40.5)
Nutritional Science	21 (9.5)
Pharmaceutical Sciences	6 (2.7)
Public Health	39 (17.6)
Grade levels, n (%)	
Freshman	36 (16.2)
Sophomore	75 (33.8)
Junior	69 (31.1)
Senior	41 (18.5)
History of psychotherapy, yes, n (%)	26 (11.7)
History of psychiatric medication, yes, n (%)	35 (15.8)
History of psychiatric disorders, yes, n (%)	42 (18.9)
Anxiety	25 (59.5)
Depression	21 (50.0)
OCD	2 (4.8)
ADHD	1 (2.4)
Panic attack disorder	1 (2.4)

(*N* = 222). SD, Standard deviation; OCD, Obsessive-Compulsive Disorder; ADHD, Attention Deficit Hyperactivity Disorder.

**Table 2:** Comparison of each variable measurements by biological sex.

Instruments	Categories	Total (*n* = 222)	Male (*n* = 41)	Female (*n* = 181)	*p*
LOT-R	Low	119 (55.3%)	18 (45.0%)	101 (57.7%)	0.06^a^
	Moderate	81 (37.7%)	16 (40%)	65 (37.1%)	
	High	15 (7.0%)	6 (15.0%)	9 (5.1%)	
	Score Mean ± S.D.	13.12 ± 3.99	14.08 ± 3.99	12.90 ± 3.97	0.09^b^
PSQI	Good	64 (29.8%)	21 (52.5)	43 (24.6%)	<0.001^a^
	Poor	151 (70.2%)	19 (47.5)	132 (75.4%)	
	Score Mean ± S.D.	7.66 ± 3.31	6.15 ± 3.17	8.00 ± 3.25	0.002^b^
PSS	Low	33 (15.3%)	13 (34.2%)	20 (11.2%)	0.001^a^
	Moderate	155 (71.8%)	23 (60.5%)	132 (74.2%)	
	High	28 (13.0%)	2 (5.3%)	26 (14.6%)	
	Score Mean ± S.D.	19.93 ± 5.86	16.55 ± 6.55	20.65 ± 5.45	<0.001^b^
GAD-7	None	59 (26.8%)	21 (52.5%)	38 (21.1%)	<0.001^a^
	Mild	61 (27.7%)	10 (25.0%)	51 (28.3%)	
	Moderate	48 (21.8%)	3 (7.5%)	45 (25%)	
	Severe	52 (23.6%)	6 (15.0%)	46 (25.6%)	
	Score Mean ± S.D.	9.25 ± 6.10	5.85 ± 5.97	10.00 ± 5.88	<0.001^b^
PHQ-9	None	80 (38.3%)	21 (56.8%)	59 (34.3%)	0.059^a^
	Mild	53 (25.4%)	10 (27.0%)	43 (25.0%)	
	Moderate	28 (13.4%)	2 (5.4%)	26 (15.1%)	
	Moderately Severe	36 (17.2%)	3 (8.1%)	33 (19.2%)	
	Severe	12 (5.7%)	1 (2.7%)	11 (6.4%)	
	Score Mean ± S.D.	8.29 ± 6.55	5.51 ± 5.48	8.88 ± 6.63	0.002^b^

SD, Standard deviation; LOT-R, revised Life Orientation Test; PSQI, Pittsburg Sleep Quality Index; PSS, Perceived Stress Scale; GAD-7, General Anxiety Disorder-7; PHQ-9, Patient Health Questionnaire-9. The *p*-values were tested using two-way-ANOVA^a^ or Mann-Whitney U test^b^.

Upon a biological sex-based analysis, it was observed that male students exhibited notably better sleep quality, lower levels of stress, anxiety, and depression in comparison to female counterparts (Table 2). However, there was no statistical difference observed in the optimism levels between male (14.08 ± 3.99) and female students (12.90 ± 3.97). In addition, no statistically significant differences were identified in the mean scores for LOT-R, PSQI, PSS, GAD-7 or PHQ-9 based on respondents’ ethnicity, study major and grade levels (Supplementary Tables 1–3).

### 3.2 Correlations of optimism with sleep quality, stress, and mental health among college students

As Table 3 shows, the college students’ optimism (LOT-R) demonstrated a significant negative correlation with poor sleep quality (*r* = −0.281, *p* < 0.001), stress (*r* = −0.486, *p* < 0.001), anxiety (*r* = −0.423, *p* < 0.001), and depression (*r* = −0.476, *p* < 0.001). Notably, poor sleep quality (PSQI >5) showed a positive correlation with stress (*r* = 0.498, *p* < 0.001), anxiety (*r* = 0.488, *p* < 0.001), and depression (*r* = 0.499, *p* < 0.001). Moreover, elevated stress levels were linked to higher anxiety (*r* = 0.675, *p* < 0.001) and depression (*r* = 0.675, *p* < 0.001).

**Table 3:** The associations of optimism with sleep quality, stress, anxiety, and depression among college students (*n* = 222).

Variables	LOT-R	PSQI	PSS	GAD-7	PHQ-9
LOT-R	1.000				
PSQI	−0.281***	1.000			
PSS	−0.486***	0.498***	1.000		
GAD-7	−0.423***	0.488***	0.675***	1.000	
PHQ-9	−0.476***	0.499***	0.675***	0.755***	1.000

LOT-R, revised Life Orientation Test; PSQI, Pittsburg Sleep Quality Index; PSS, Perceived Stress Scale; GAD-7, General Anxiety Disorder-7; PHQ-9, Patient Health Questionnaire 9. Pearson’s Correlation, ***p < 0.001.

### 3.3 The mediating role of stress and sleep quality

#### 3.3.1 Parallel mediation models

**Figure 2:**
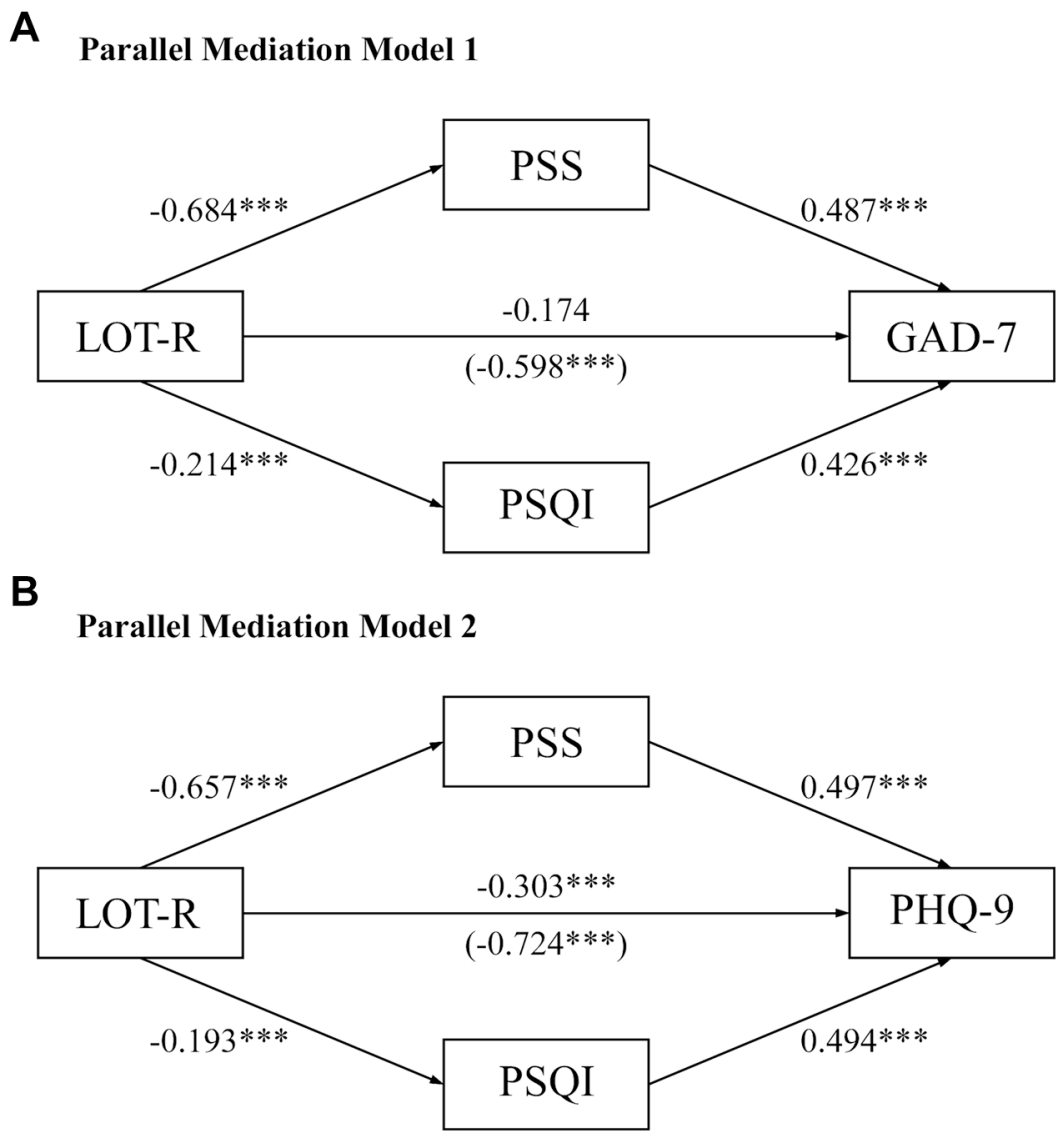
Parallel mediation models for the mediating effects of stress and sleep quality on the relationship between optimism and mental health. Direct and indirect effects of optimism on **(A)** anxiety or **(B)** depression through stress and sleep quality are illustrated. The models are adjusted for biological sex. All effects presented are unstandardized. The direct effect and the (total effect) are depicted on the path directly from optimism to anxiety or depression. ***p < 0.001. LOT-R, revised Life Orientation Test; PSQI, Pittsburg Sleep Quality Index; PSS, Perceived Stress Scale; GAD-7, General Anxiety Disorder-7; PHQ-9, Patient Health Questionnaire-9.

To understand how optimism, directly and indirectly, influenced anxiety or depression, parallel mediation models were conducted. In Figure 2A, as the direct effect of optimism toward anxiety became insignificant (Direct Effect [DE] = −0.174, *p* = 0.060), stress and sleep quality fully mediated the association between optimism and anxiety after the adjustment for biological sex. Specifically, stress and sleep quality exhibited significant indirect effects on this association (Indirect Effect [IE]_PSS_ = −0.333, *p* < 0.001; IE_PSQI_ = −0.091, *p* = 0.005). These findings indicated that students with enhanced optimism may experience lower anxiety levels through reduced stress and improved sleep quality. Besides, compared to sleep quality, lower perceived stress was the more dominant route on the impact of optimism on anxiety.

Following the adjustment for biological sex and the introduction of stress and sleep quality into the Model 2 (Figure 2B), the attenuated path coefficient (DE = −0.303, *p* = 0.001) of optimism on depression revealed the partial mediating roles of stress and sleep quality in the optimism-depression relationship. Both stress and sleep quality demonstrated significant mediating effects on optimism with depression (IE_PSS_ = −0.327, *p* < 0.001; IE_PSQI_ = −0.095, *p* = 0.015), reflecting the stronger mediating role of stress. Similar to the Model 1 (Figure 2A), students with higher optimism levels were less susceptible to depression through reduced stress and enhanced sleep quality. Additionally, the influence of optimism on depression appeared to be primarily channeled through lower perceived stress. Overall, the total effects derived from these two models revealed statistically significant negative associations between optimism and anxiety (β_total_ = −0.598, 95% CI: −0.778 to −0.392), and optimism and depression (β_total_ = −0.724, 95% CI: −0.919 to −0.519), which implied a more pronounced impact of optimism on depression compared to anxiety.

#### 3.3.2 Serial mediation models

**Figure 3:**
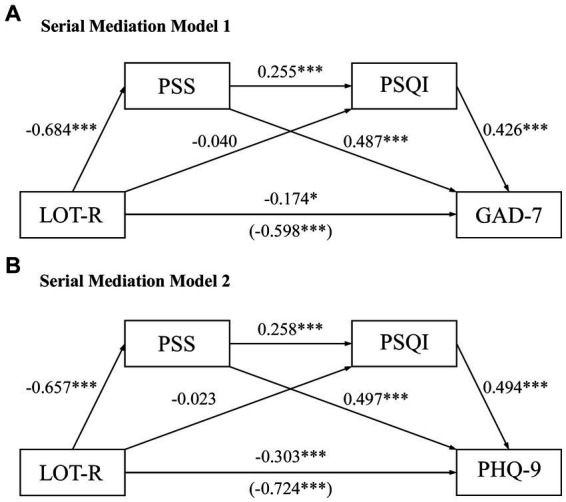
Serial mediation models for the mediating effects of stress and sleep quality on the relationship between optimism and mental health. Four serial mediating pathways are illustrated, respectively, in the serial mediation models of **(A)** optimism to anxiety and **(B)** optimism to depression. The models are adjusted for biological sex. All effects presented are unstandardized. The direct effect and the (total effect) are depicted on the path directly from optimism to anxiety or depression. *p < 0.05, ***p < 0.001. LOT-R, revised Life Orientation Test; PSQI, Pittsburg Sleep Quality Index; PSS, Perceived Stress Scale; GAD-7, General Anxiety Disorder-7; PHQ-9, Patient Health Questionnaire-9.

To explore the mediating roles of stress and sleep quality linked in casual chains, guided by a specific directional flow, serial mediation models were employed. Since two mediators of stress and sleep quality were used and with two different outcome variables (anxiety and depression), a total of four different causal order models were produced, with only two of which were presented in Figure 3. Each distinct casual order of the mediators was examined to compare the significant paths generated by the four models. The total indirect effects derived from serial mediation models were all found to be statistically significant. Of note, the two mediators were shown to partially mediate in the relationship between optimism and anxiety, as well as in the optimism-depression link. This observation denotes a nuanced departure from the result derived from the parallel mediation models (Figure 2).

In the analysis of serial mediation models depicting the transition from optimism to anxiety, both the indicated indirect effect path (LOT-R/PSS/PSQI/GAD-7, IE = −0.074, 95% CI: −0.135 to −0.029) presented in Figure 3A and the alternative indirect effect path (LOT-R/PSQI/PSS/GAD-7, IE = −0.066, 95% CI: −0.114 to −0.032) demonstrated statistical significance. Additionally, within the serial mediation models that trace the impact of optimism on depression, the specified indirect pathway denoted as LOT-R/PSS/PSQI/PHQ-9 (Figure 3B, IE = −0.084, 95% CI: −0.142 to −0.036) and the alternative pathway of LOT-R/PSQI/PSS/PHQ-9 (IE = −0.064, 95% CI: −0.114 to −0.026) similarly exhibited significant mediating effect. Collectively, these findings imply that increased optimism would lead to lower perceived stress, subsequently improving sleep quality and ultimately contributing to reduced levels of anxiety and depression.

## 4 Discussion

This study explored the inter-relations between optimism and mental health in college students, with a specific aim to examine the mediating effects of sleep quality and stress in the relationship. Our findings revealed a significant negative correlation between optimism and poor sleep quality, stress, anxiety as well as depression. The result was consistent with the previous finding indicating that college students with higher scores on optimism reported improved sleep quality and lower levels of stress, anxiety, and depression, which might be targeted to reduce mental health problems and improve academic success (Cheuk Yan and Wong, 2011; Kim et al., 2022). Additionally, parallel mediation analysis demonstrated that stress and sleep quality fully mediated the relationship between optimism and anxiety, while partially mediating the optimism-depression link. Regarding serial mediation analysis, a significant mediating path sequentially followed by stress and sleep quality was demonstrated both in optimism-anxiety and optimism-depression models.

With the potential underlying mechanism inferred from parallel and serial mediation analyses, this study highlighted the importance of optimism as a mechanism through which reduced levels of stress and improved sleep quality can translate into anxiety and depression. Moreover, the findings also underlined the vulnerability of health science students, as they contend with a variety of academic and clinical stressors, including long hours of study, the demanding nature of examinations, and lack of free time (Papazisis et al., 2008; Sakai et al., 2022). Therefore, school-based interventions may hold promise in ameliorating students’ stress, improving their sleep quality, and further reducing the levels of anxiety, and depression.

Aside from examining the association between optimism and mental health among college students, this study also serves to confirm the degree of optimism, sleep quality, stress, anxiety, and depression experienced by college students in the post-COVID era. The emergence of the coronavirus disease 2019 (COVID-19) has brought forth not only physical health problems but also psychological issues (Tran et al., 2020; Xiong et al., 2020). College students, in particular, are adversely impacted owing to the uncertainty surrounding academic achievement, future career prospects, and social lives (Aristovnik et al., 2021). The disruptions caused by school closures, cancelation of social events, remote online courses, and exam postponements during the COVID-19 pandemic heightened their emotional distress (Cao et al., 2020; Lei et al., 2020; Xiong et al., 2020). However, the psychological challenges faced by students did not end with the remission of the pandemic and the easing of social restrictions. In this study, our participants reported low optimism, poor sleep quality, moderate stress, mild anxiety, and depression, reflecting the persisting psychological impact of the pandemic. Consistent with our findings, other study has also highlighted that students perceived intensified levels of stress and anxiety, as well as moderate depression after returning to campus (Al-Rawi et al., 2022). Factors contributing to their psychological struggles include fear of infection and enduring social, family, and economic changes resulting from the COVID-19 pandemic (Wang et al., 2022).

There are several limitations inherent in this study. First, the study sample was exclusively drawn from Health Sciences students at a single public university in the United States, thereby limiting the generalizability of the findings to broader populations or countries. Second, the biological sex composition of the participants was predominately female, and recent studies have shown biological sex-based differences in student mental health consequences of the COVID-19 pandemic (Ausín et al., 2021; Prowse et al., 2021). Hence, this biological sex imbalance may limit the generalizability of the results to the male population. Third, the study lacked longitudinal follow-up and, therefore, did not demonstrate causal relationships, although the outcomes do imply that researchers, clinicians, and schools should take into account these variable interactions between optimism and mental health among college students.

## 5 Conclusion

This study documented the direct and indirect effects of stress and sleep quality and its sequential mediating pathway in the connection between optimism and mental health within health science college students. Findings from the study underscore the significance of fostering academic optimism to alleviate stress and improve sleep quality, ultimately expecting to ease the mental health burdens experienced by college students. Consequently, the development of diverse academic programs focused on enhancing the optimism of college students becomes imperative.

## Data availability statement

The raw data supporting the conclusions of this article will be made available by the authors, without undue reservation.

## Ethics statement

The study involving human participants was reviewed and approved by the Institutional Review Board (IRB) at the University of Massachusetts Lowell (IRB# 22-072-LAI-EXM). The studies were conducted in accordance with the local legislation and institutional requirements. The participants provided their written informed consent to participate in this study.

## Author contributions

Y-JL: Conceptualization, Data curation, Formal analysis, Funding acquisition, Investigation, Methodology, Project administration, Resources, Supervision, Validation, Visualization, Writing – original draft, Writing – review & editing. E-YT: Resources, Visualization, Writing – review & editing. PJ: Formal analysis, Writing – original draft, Writing – review & editing. Y-SW: Formal analysis, Methodology, Writing – review & editing. Y-HC: Formal analysis, Methodology, Writing – review & editing. SO’L: Data curation, Writing – review & editing. SM: Data curation, Writing – review & editing. YZ: Conceptualization, Validation, Writing – review & editing. JS: Validation, Writing – review & editing. YW: Methodology, Validation, Writing – review & editing.
