# Supplementary material for: Optimism and mental health in college students: the mediating role of sleep quality and stress
Source: Front Psychol. 2024 Jul 16;15:1403146. doi: 10.3389/fpsyg.2024.1403146 (PMC11286569; doi:10.3389/fpsyg.2024.1403146)
Supplement: Supplementary file 1 [file Table_1.docx]

| **Supplementary Table 1: The associations of race/ethnicity with optimism, sleep quality, stress, anxiety, and depression among college students** | | | | |
| --- | --- | --- | --- | --- |
| **Variables** | **Race/Ethnicity** | **N** | **Mean Rank** | **P-value** |
| **LOT-R**  (N = 215) | Non-Hispanic White or European-American | 95 | 112.59 | 0.642 |
|  | African American, Afro-Caribbean | 28 | 113.73 |  |
|  | Latino or Hispanic American | 41 | 107.49 |  |
|  | Asian and Asian American | 49 | 96.28 |  |
|  | Others | 2 | 107.25 |  |
| **PSQI**  (N = 215) | Non-Hispanic White or European-American | 94 | 101.85 | 0.382 |
|  | African American, Afro-Caribbean | 28 | 110.96 |  |
|  | Latino or Hispanic American | 42 | 124.35 |  |
|  | Asian and Asian American | 49 | 103.76 |  |
|  | Others | 2 | 116.25 |  |
| **PSS**  (N = 216) | Non-Hispanic White or European-American | 97 | 106.33 | 0.893 |
|  | African American, Afro-Caribbean | 29 | 112.36 |  |
|  | Latino or Hispanic American | 41 | 110.18 |  |
|  | Asian and Asian American | 47 | 107.41 |  |
|  | Others | 2 | 148.75 |  |
| **GAD-7**  (N = 220) | Non-Hispanic White or European-American | 97 | 111.26 | 0.971 |
|  | African American, Afro-Caribbean | 28 | 103.80 |  |
|  | Latino or Hispanic American | 43 | 110.16 |  |
|  | Asian and Asian American | 50 | 113.58 |  |
|  | Others | 2 | 97.50 |  |
| **PHQ-9**  (N = 209) | Non-Hispanic White or European-American | 96 | 104.74 | 0.572 |
|  | African American, Afro-Caribbean | 25 | 87.22 |  |
|  | Latino or Hispanic American | 42 | 110.90 |  |
|  | Asian and Asian American | 44 | 110.23 |  |
|  | Others | 2 | 100.50 |  |
| LOT-R, revised Life Orientation Test; PSQI, Pittsburg Sleep Quality Index; PSS, Perceived Stress Scale; GAD-7, General Anxiety Disorder-7; PHQ-9, Patient Health Questionnaire 9.  Kruskal-Wallis Test, Sig. p < 0.05 | | | | |
