# Supplementary material for: Optimism and mental health in college students: the mediating role of sleep quality and stress
Source: Front Psychol. 2024 Jul 16;15:1403146. doi: 10.3389/fpsyg.2024.1403146 (PMC11286569; doi:10.3389/fpsyg.2024.1403146)
Supplement: Supplementary file 2 [file Table_2.docx]

| **Supplementary Table 2: The associations of study major with optimism, sleep quality, stress, anxiety, and depression among college students** | | | | | |
| --- | --- | --- | --- | --- | --- |
| **Variables** | **Study Major** | **N** | **Mean Rank** | **P-value** | |
| **LOT-R**  (N = 215) | Biomedical Sciences | 35 | 103.17 | 0.506 | |
|  | Exercise Science | 28 | 95.86 |  |  |
|  | Nursing | 89 | 111.13 |  |  |
|  | Nutritional Science | 21 | 127.62 |  |  |
|  | Pharmaceutical Sciences | 5 | 121.80 |  |  |
|  | Public Health | 37 | 101.23 |  |  |
| **PSQI**  (N = 215) | Biomedical Sciences | 34 | 128.07 | 0.148 | |
|  | Exercise Science | 28 | 110.71 |  |  |
|  | Nursing | 89 | 101.24 |  |  |
|  | Nutritional Science | 20 | 89.10 |  |  |
|  | Pharmaceutical Sciences | 6 | 140.00 |  |  |
|  | Public Health | 38 | 108.76 |  |  |
| **PSS**  (N = 216) | Biomedical Sciences | 36 | 130.07 | 0.311 | |
|  | Exercise Science | 29 | 104.81 |  |  |
|  | Nursing | 87 | 107.61 |  |  |
|  | Nutritional Science | 21 | 95.60 |  |  |
|  | Pharmaceutical Sciences | 6 | 103.33 |  |  |
|  | Public Health | 37 | 100.65 |  |  |
| **GAD-7**  (N = 220) | Biomedical Sciences | 37 | 120.95 | 0.318 | |
|  | Exercise Science | 28 | 108.09 |  |  |
|  | Nursing | 89 | 115.63 |  |  |
|  | Nutritional Science | 21 | 83.05 |  |  |
|  | Pharmaceutical Sciences | 6 | 97.67 |  |  |
|  | Public Health | 39 | 107.36 |  |  |
| **PHQ-9**  (N = 209) | Biomedical Sciences | 35 | 112.80 | 0.666 | |
|  | Exercise Science | 25 | 112.84 |  |  |
|  | Nursing | 88 | 105.36 |  |  |
|  | Nutritional Science | 21 | 85.83 |  |  |
|  | Pharmaceutical Sciences | 5 | 98.10 |  |  |
|  | Public Health | 35 | 103.19 |  |  |
| LOT-R, revised Life Orientation Test; PSQI, Pittsburg Sleep Quality Index; PSS, Perceived Stress Scale; GAD-7, General Anxiety Disorder-7; PHQ-9, Patient Health Questionnaire 9. Kruskal-Wallis Test, Sig. p < 0.05 | | | | |  |
