# Supplementary material for: Optimism and mental health in college students: the mediating role of sleep quality and stress
Source: Front Psychol. 2024 Jul 16;15:1403146. doi: 10.3389/fpsyg.2024.1403146 (PMC11286569; doi:10.3389/fpsyg.2024.1403146)
Supplement: Supplementary file 3 [file Table_3.docx]

| **Supplementary Table 3: The associations of grade levels with optimism, sleep quality, stress, anxiety, and depression among college students** | | | | |
| --- | --- | --- | --- | --- |
| **Variables** | **Grade Levels** | **N** | **Mean Rank** | **P-value** |
| **LOT-R**  (N = 214) | Freshman | 36 | 111.03 | 0.113 |
|  | Sophomore | 72 | 103.16 |  |
|  | Junior | 66 | 98.39 |  |
|  | Senior | 40 | 127.16 |  |
| **PSQI**  (N = 214) | Freshman | 34 | 103.38 | 0.939 |
|  | Sophomore | 75 | 108.24 |  |
|  | Junior | 67 | 110.45 |  |
|  | Senior | 38 | 104.53 |  |
| **PSS**  (N = 215) | Freshman | 34 | 107.31 | 0.401 |
|  | Sophomore | 74 | 110.57 |  |
|  | Junior | 68 | 113.96 |  |
|  | Senior | 39 | 93.32 |  |
| **GAD-7**  (N = 219) | Freshman | 35 | 125.74 | 0.090 |
|  | Sophomore | 75 | 114.50 |  |
|  | Junior | 69 | 108.59 |  |
|  | Senior | 40 | 90.23 |  |
| **PHQ-9**  (N = 208) | Freshman | 34 | 109.74 | 0.192 |
|  | Sophomore | 71 | 104.48 |  |
|  | Junior | 66 | 112.08 |  |
|  | Senior | 37 | 86.20 |  |
| LOT-R, revised Life Orientation Test; PSQI, Pittsburg Sleep Quality Index; PSS, Perceived Stress Scale; GAD-7, General Anxiety Disorder-7; PHQ-9, Patient Health Questionnaire 9. Kruskal-Wallis Test, Sig. p < 0.05 | | | | |
